# A promising gene delivery system developed from PEGylated MoS_2_ nanosheets for gene therapy

**DOI:** 10.1186/1556-276X-9-587

**Published:** 2014-10-27

**Authors:** Zhongyang Kou, Xin Wang, Renshun Yuan, Huabin Chen, Qiaoming Zhi, Ling Gao, Bin Wang, Zhaoji Guo, Xiaofeng Xue, Wei Cao, Liang Guo

**Affiliations:** 1 Department of General Surgery, the First Affiliated Hospital of Soochow University, 188 Shizi Road, Suzhou 215006, China; 2 Department of Radiology, the First Affiliated Hospital of Soochow University, 188 Shizi Road, Suzhou 215006, China

**Keywords:** Two-dimensional (2D) nanomaterial, MoS_2_ nanosheet, RNA interference

## Abstract

A new class of two-dimensional (2D) nanomaterial, transition metal dichalcogenides (TMDCs) such as MoS_2_, MoSe_2_, WS_2_, and WSe_2_ which have fantastic physical and chemical properties, has drawn tremendous attention in different fields recently. Herein, we for the first time take advantage of the great potential of MoS_2_ with well-engineered surface as a novel type of 2D nanocarriers for gene delivery and therapy of cancer. In our system, positively charged MoS_2_-PEG-PEI is synthesized with lipoic acid-modified polyethylene glycol (LA-PEG) and branched polyethylenimine (PEI). The amino end of positively charged nanomaterials can bind to the negatively charged small interfering RNA (siRNA). After detection of physical and chemical characteristics of the nanomaterial, cell toxicity was evaluated by 3-(4,5-dimethylthiazol-2-yl)-2,5-diphenyltetrazolium bromide (MTT) assay. Polo-like kinase 1 (PLK1) was investigated as a well-known oncogene, which was a critical regulator of cell cycle transmission at multiple levels. Through knockdown of PLK1 with siRNA carried by novel nanovector, qPCR and Western blot were used to measure the interfering efficiency; apoptosis assay was used to detect the transfection effect of PLK1. All results showed that the novel nanocarrier revealed good biocompatibility, reduced cytotoxicity, as well as high gene-carrying ability without serum interference, thus would have great potential for gene delivery and therapy.

## Background

RNA interference (RNAi) is a newly discovered cellular strategy for silencing genes in a sequence-specific manner [1-3]. At present, small interfering RNA (siRNA)-mediated gene regulation or therapy has shown immense potential in treating various diseases by silencing abnormally up-regulated genes [4-6]. In this kind of gene therapy, siRNA could be delivered into cells utilizing either viral vectors or non-viral carriers, causing degradation of targeted mRNA and subsequently leading to the silence of specific protein expression [7]. The success of gene therapy is largely dependent on the development of a safe and efficient gene delivery system [8-10]. Viral vectors have been primarily used in gene therapy due to their high delivery efficiency [11,12]. However, despite of the advantages such as simplicity of use, ease of large-scale production, and lack of specific immune response [13,14], viral vectors might bring the side effects such as endogenous virus recombination, oncogenic effects, and unexpected immune response [15] while non-viral vectors could circumvent. Many non-viral-based gene delivery vectors, such as cationic polymers [16], silica nanoparticles [17], iron oxide nanoparticles [18], and many other types [19-21], have been extensively explored in recent years. Though they are widely used for research in gene delivery, their toxicity and low *in vivo* efficiency limited their further application [22].

Thus, seeking for new biological materials has become an important research direction [23]. In recent years, nanoparticles have demonstrated unique physical and biological properties that can be applied to overcome the issues in gene and drug delivery systems due to its superior characteristics [24]. For example, nanoparticle size is usually 10 to 100 nm, which is capable of penetrating through the submucosal layers and enhances the efficiency in gene transfection level [25]. Besides, a number of cationic polymers have been investigated as gene carriers, such as polyethylenimine (PEI) due to its specific features. PEI has high pH-buffering capacity, lower cytotoxicity, and high transfection efficiency [26], thus has tremendous potential in gene therapy.

The novel nanomaterials, which could be developed from graphene or transition metal dichalcogenides (TMDCs) such as MoS_2_, MoSe_2_, WS_2_, and WSe_2_, came to be new emerging non-viral gene delivery carriers [27-30]. Graphene and its analog, TMDCs, which are the two-dimensional (2D) sp^2^-bonded nanocarbon with excellent electronic, optical, and mechanical properties have been extensively studied in the past decades [31,32].

In this study, MoS_2_ was formulated as nanoparticles and modified by PEI on the particles to increase the surface charge, providing as a promising gene carrier candidate. The obtained positively charged MoS_2_-PEG-PEI could be loaded with siRNA for gene delivery. Our results for the first time suggested TMDCs as a novel type of 2D nanovector in gene delivery with low cytotoxicity and high transfection efficiency without serum interference, promising for future applications in non-viral based gene therapy.

## Methods

### Materials

Branched polyethylenimine (PEI) with molecular weight (MW) of 25 kDa and 3-(4,5-dimethylthiazol-2-yl)-2,5-diphenyltetrazolium bromide (MTT) were obtained from Sigma-Aldrich (St. Louis, MO, USA). Lipoic acid-modified polyethylene glycol (LA-PEG) polymers were purchased from PegBio (Suzhou, China). Lipofectamine 2000 transfection kit, 4′,6′-diamidino-2-phenylindole (DAPI), and fetal bovine serum (FBS) were obtained from Invitrogen (Carlsbad, CA, USA). Dulbecco’s modified Eagle’s medium (DMEM) was purchased from Thermo Scientific (Waltham, MA, USA). SiRNA-targeting polo-like kinase 1 (PLK1) gene and negative control siRNA with a scrambled sequence were synthesized with fluorescent label by GenePharma Co., LTD (Suzhou, China). Sequence was as follows: siPLK1, 5′-AUAUUCGA CUUUGGUUGCCdTdT-3′, siN.C., 5′-ACGUGACAC GUUCGGAGAAdTdT-3′. The entire antibodies were supplied by Abcam Co., LTD (Cambridge, MA, USA).

### Synthesis of single-layer MoS_2_ nanosheets

MoS_2_ nanosheets were synthesized by the Morrison method [33]. Shortly, 500 μg MoS_2_ crystal was soaked in 500 μL of 1.6 M n-butyllithium solution in hexane for 2 days inside a nitrogen glove box. Following the intercalation by lithium, the MoS_2_ sample was filtered and washed repeatedly with 80 mL hexane to remove excess lithium and other organic residues. Intercalated MoS_2_ sample was then removed immediately from glove box and ultrasonicated in water for 1 h to allow effective exfoliation, obtaining exfoliated MoS_2_ which was then centrifuged in 3,000 rpm to remove unexfoliated MoS_2_ and excess LiOH in the precipitates. The supernatant was dialyzed against deionized water using membranes with molecular weight cut-off (MWCO) of 14 kDa for 2 days to remove lithium compounds and other residue ions, obtaining MoS_2_ nanosheets dispersed in water for future use.

### PEGylation of MoS_2_ nanosheets and preparation of MoS_2_-PEG-PEI

Ten milligrams of lipoic acid-modified PEG (LA-PEG) was added into 1 mg of MoS_2_ nanosheets dispersed in 2 mL of water. After sonication for 20 min and stirring overnight, excess PEG polymers were removed by centrifugal filtration with 100 kDa MWCO filters (Millipore, Billerica, MA, USA) and several times of water washing. The obtained MoS_2_-PEG or MoS_2_-PEG-FA were highly water-soluble and stored less than 4°C for use.

Generally, PEI used during this experiment was pre-dissolved in deionized water. One milligram of PEGylated MoS_2_ nanosheets and 0.1 mL PEI (50 mg/mL) were mixed in 2 mL deionized water. The mixture was stirred overnight under room temperature. Free PEI was removed by hyperfiltration.

### Characterization of the prepared nanomaterials

Atomic force microscopy (AFM) (Veeco Inc., Plainview, NY, USA) was used to characterize the size and thickness of MoS_2_ nanosheets before and after PEG coating. Elemental analysis data were acquired by an elemental analyzer (EA1110 CHNO-S, Carlo Erba, Cornaredo, MI, Italy). Zeta potentials and size distributions of nanoparticles were measured by a Nano-ZS90 nanoparticle analyzer (Malvern Instruments Ltd.).

### Loading of siRNA onto MoS_2_-PEG-PEI and agarose gel electrophoresis assay

From elemental analysis (nitrogen content), we estimated that the PEI content in MoS_2_-PEG-PEI conjugate was about 32%. MoS_2_-PEG-PEI was mixed with 20 pmol siRNA in 20 μL deionized water at different nitrogen/phosphor (N/P) ratios (N/P =0, 5, 10, 15, 20). The mixtures were then incubated for 1 h at room temperature before they were analyzed by 1% agarose gel electrophoresis in Tris-acetate-EDTA (TAE) buffer.

### Cellular experiments

HepG2 cell line obtained from American Type Culture Collection (ATCC, Manassas, VA, USA) was cultured in Dulbecco’s modified Eagle’s medium (DMEM) containing 10% FBS and 1% penicillin/streptomycin at 37°C in a humidified 5% CO_2_-containing atmosphere.

For siRNA transfection, HepG2 cells were seeded in 35 mm culture dishes at a density of 1 × 10^5^ cells per well. We diluted 200 pmol FAM-siRNA in 200 μL serum-containing DMEM and various concentrations of MoS_2_-PEG-PEI in 200-μL serum-containing DMEM. The two solutions were mixed together and incubated for 20 min at room temperature before being added into cells, maintaining the final volume at 2 mL. Here, we used Lipofectamine 2000 as the positive transfection agent and siRNA with a scrambled sequence as the negative control. After 6 h of transfection, cells were washed twice with PBS (pH = 7.4) and then imaged by a laser scanning confocal microscope (Leica SP5, Leica Microsystems, Wetzlar, Germany). The cell nuclei could be stained by DAPI. After 6 h siPLK1 without FAM label transfection following the same protocol, we transfered the cells into new fresh complete media and incubated at 37°C for additional 48 h. Cells could be stained with the calcein-AM/propidium iodide (PI) to determine the viability.

### RNA extraction and quantitative real-time PCR (qPCR)

All transfected cells were washed twice with PBS and the total RNA was extracted using the TRIzol reagent (Takara, Dalian, China) according to the manufacturer’s protocol. Then, RNA was subsequently reversely transcribed to complementary DNA (cDNA) using M-MLV First-Strand cDNA Synthesis Kit (Invitrogen, Carlsbad, CA, USA). Afterwards, qPCR analysis was performed using Platinum SYBR Green qPCR SuperMix-UDG kits (Invitrogen, Carlsbad, CA, USA) on an ABI Prism 7500 Real-Time PCR system (Applied Biosystems, Foster City, CA, USA).The relative amount of PLK1 normalized to β-actin was calculated according to the 2^−∆∆Ct^ method. Each sample was run in triplicate. The primer sequences were as follows: 5′-AGCCTGAGGCCCGATACTACCTAC-3′ (PLK1-forward), 5′-ATTAGGAGTCCCACACAGGGTCTTC-3′ (PLK1-reverse) and 5′-GCACAGAGCCTCGCCTT-3′ (β-actin-forward), 5′-GTTGTCGACGACGAGCG-3′ (β-actin -reverse).

### Western blotting analysis

Total proteins were prepared by standard procedures and quantified by the Bradford BSA Protein Assay Kit. Equivalent amounts of protein were resolved and mixed with loading buffer, then loaded on 10% SDS-PAGE gel and subsequently electrotransferred to a polyvinylidene difluoride (PVDF) membrane, which was blocked for 2 h at room temperature with 5% non-fat dry milk in PBS. After blocking, the membrane was incubated with mouse anti-human PLK1 antibody at 1:1,000 at 4°C overnight followed by incubation with goat-anti-mouse IgG antibody at 1:2,000 for 1 h at room temperature. Mouse-anti-human β-actin antibody diluted at 1:1,000 was used as a control. Densitometric values of protein bands were quantified using Image Analysis software on Evolve-512 photometric system.

### Flow cytometry analysis

Flow cytometry analysis was employed to quantify the cell apoptosis post treatment by using Annexin V-FITC/PI apoptosis detection kit. In details, 24 h after transfection, cells were detached by trypsin, washed with PBS, and then re-suspended in 500 μL Annexin V binding buffer containing 1 μg/L Annexin V-FITC and 4 μg/L PI. The samples were then analyzed by flow cytometry (BD FACS Calibur, BD Biosciences, San Jose, CA, USA).

### Statistical analysis

Statistical analysis was performed using SPSS15.0 software. Data are expressed as the mean ± standard deviation from at least three separate experiments. Differences between groups were analyzed using Student’s *t*-test. A value of *p* <0.05 was considered statistically significant.

## Results and discussion

**Figure 1:**
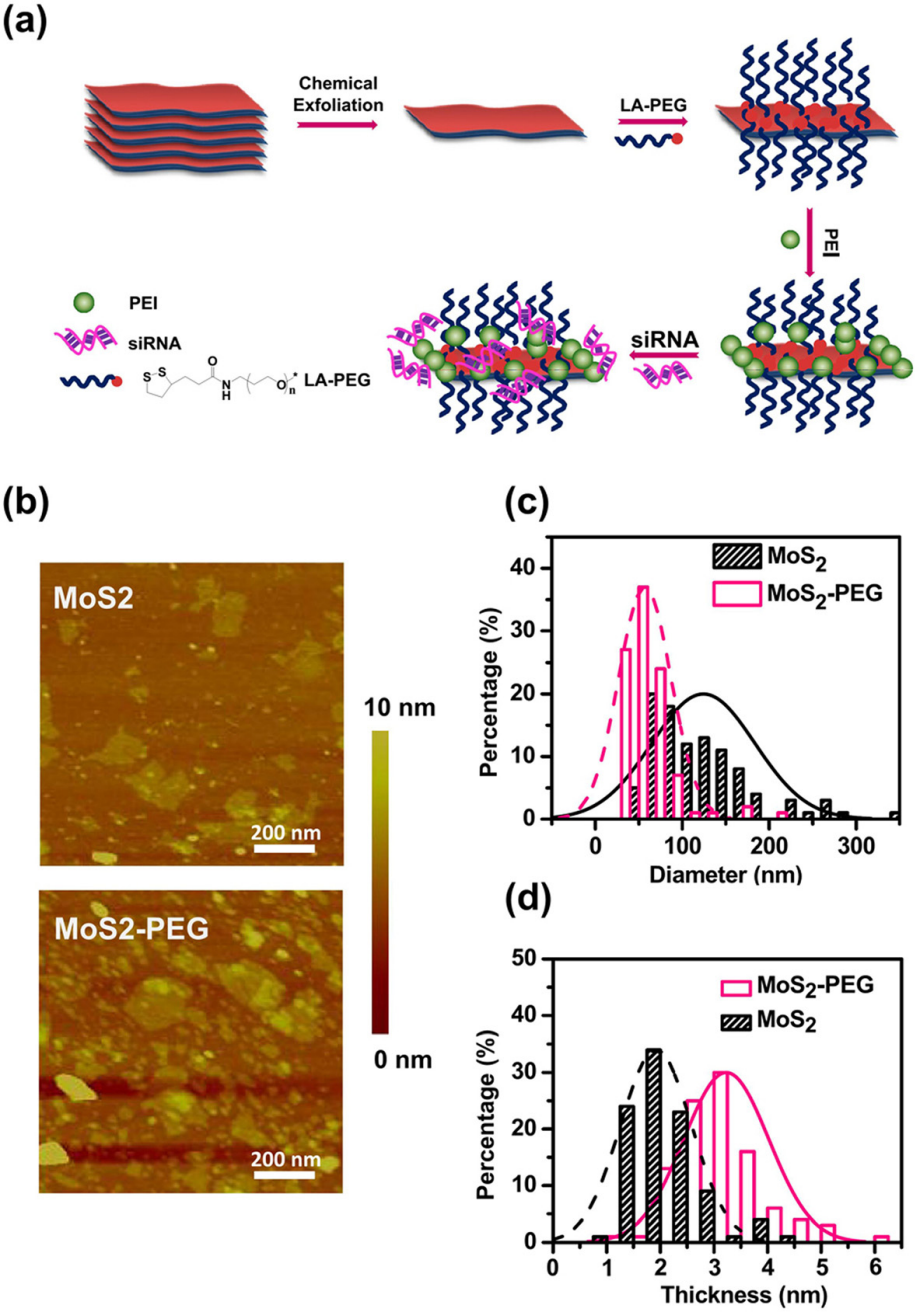
**Synthesis and characterization of MoS**_**2 **_**and MoS**_**2**_**-PEG. (a)** A scheme showing the preparation of MoS_2_-PEG-PEI and the subsequent loading with siRNA. **(b)** AFM images of MoS_2_ before and after PEGylation. **(c, d)** AFM measured diameter (c) and thickness (d) distributions of MoS_2_ and MoS_2_-PEG. Over 100 nanosheets were counted for each sample.

The schematic illustration to show the construction of MoS_2_-PEG-PEI/siRNA is shown in Figure 1a. Two-dimensional MoS_2_ nanosheets were prepared by the chemical exfoliation method according to the literature [34]. To enhance the stability of MoS_2_ in serum, the as-made single-layer MoS_2_ nanosheets were then conjugated the lipoic acid-modified PEG (LA-PEG) which is well-known for its excellent ability to prevent non-specific binding of proteins on nanomaterial surface together with the branched polyethylenimine (PEI) polymer. From elemental analysis (nitrogen content), we estimated that the PEI content in MoS_2_-PEG-PEI conjugate was about 32%. According to the previous studies [28,34,35], we used the thiol chemistry method to functionalize MoS_2_ nanosheets by coating the surface of MoS_2_ with LA-PEG which contained a disulfide group on the PEG terminal. Furthermore, the obtained positively charged MoS_2_-PEG-PEI could be loaded with siRNA which was negatively charged for gene delivery. To improve the physiological stability and biocompatibility of MoS_2_ nanosheets, surface modification of the 2D nanosheets was required before using them for bioapplications. MoS_2_-PEG-PEI showed excellent stability in both saline and serum-containing cell medium at room temperature (Additional file 1: Figure S1a). Consistent to this observation, MoS_2_-PEG-PEI kept their consistent hydrodynamic sizes at about 50 nm in the serum-containing cell medium (Additional file 1: Figure S1b). The great stability of MoS_2_-PEG-PEI in the presence of serum makes it a promising candidate for gene delivery without serum interference.

**Figure 2:**
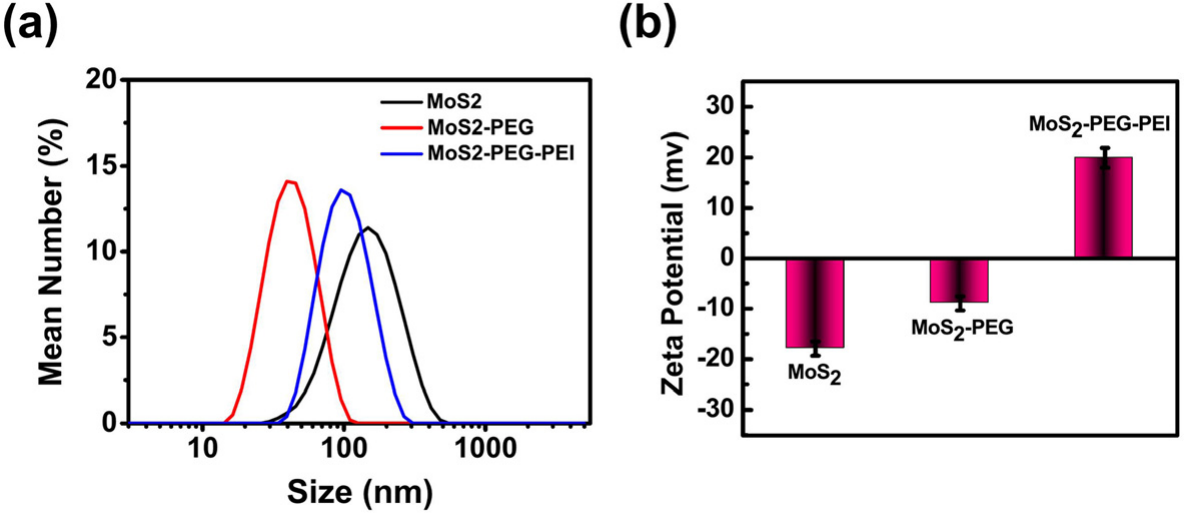
**Characterization of different layers of MoS**_**2 **_**nanosheets. (a)** Hydrodynamic sizes of MoS_2,_ PEGylated MoS_2_, and MoS_2_-PEG-PEI. **(b)** Zeta potentials of MoS_2_ nanosheets before and after two layers of polymer coatings measured in water.

AFM was used to characterize MoS_2_ nanosheets before and after PEGylation (Figure 1b). It revealed that the original chemically-exfoliated MoS_2_ nanosheets showed an average diameter of ∼ 100 nm and an average thickness of ∼ 1.8 nm. After the LA-PEG coating, the average diameter of MoS_2_ nanosheets decreased to ∼ 50 nm because ultrasonication step might partially break down those nanosheets. However, the average thickness of PEGylated MoS_2_ increased to ∼ 2.8 nm, owing to the existence of PEGylation (Figure 1c). The size distributions of MoS_2_ before and after PEG coating are suggested by AFM images and dynamic light scattering (DLS) data in the meantime (Figures 1c and 2a).

We then evaluated the size distributions and zeta potentials of different layers of MoS_2_-PEG-PEI (Figure 2a,b). The zeta potentials of MoS_2_ and MoS_2_-PEG were measured to be −17.9 and −8.9 mV, respectively. MoS_2_-PEG-PEI with positively charged PEI coating showed an increased zeta potential of 19.9 mV. DLS data revealed that MoS_2_-PEG had much smaller sizes compared to original MoS_2_ nanosheets. And the size of MoS_2_-PEG-PEI was between MoS_2_ and MoS_2_-PEG.

Before we used the MoS_2_ nanosheets in *in vitro* drug delivery experiments, we tested their potential toxicity by the MTT assay which was performed to determine the relative cell viability. HepG2, HeLa, and 293 T cells were respectively incubated with various concentrations of MoS_2_, MoS_2_-PEG, and MoS_2_-PEG-PEI for 24 h.

**Figure 3:**
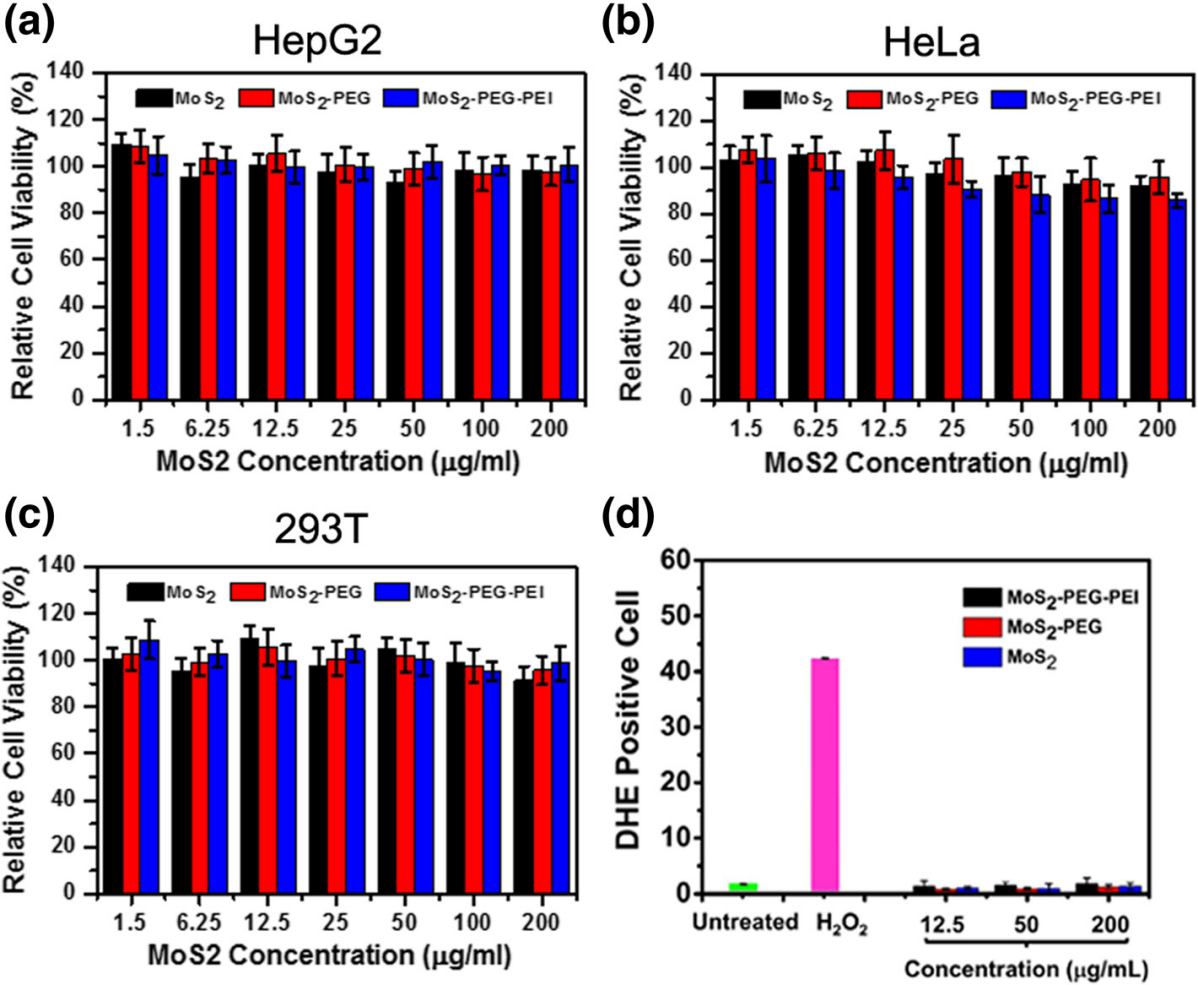
***In vitro *cell toxicity assay of different layers of MoS**_**2 **_**nanosheets.** Relative viabilities of HepG2 **(a)**, HeLa **(b)**, and 293 T **(c)** cells determined by the MTT assay after incubation with various concentrations of MoS_2,_ MoS_2_-PEG, and MoS_2_-PEG-PEI for 24 h. **(d)** Percentages of DHE-positive cells (cells with significant oxidative stress) after incubation with various concentrations of MoS_2_, MoS_2_-PEG, and MoS_2_-PEG-PEI for 24 h. H_2_O_2_ (200 μM) incubated cells were used as the control. Error bars were based on four parallel samples.

In general, we found that there was no significant cytotoxicity about MoS_2_, PEGylated MoS_2_, and MoS_2_-PEG-PEI (Figure 3a-c). Three kinds of cells survived after 24 h incubation even under the high concentration of MoS_2_ up to 0.2 mg/mL. Cells which were incubated with MoS_2_-PEG showed higher viability compared to those which were incubated with plain MoS_2._ However, MoS_2_-PEG-PEI exhibited slightly reduced viability mainly because it was positively charged.

The above result obviously displayed that the cytotoxicity of MoS_2_ nanosheets, like many other nanomaterials, was closely related to their surface chemistry. Therefore, well-designed surface modification plays an important role in the biomedical application of this type of 2D nanomaterials.

Previous studies revealed that many nanoparticles could generate reactive oxygen species (ROS) such as •O^2−^, •OH, and H_2_O_2_ in the exposed cells, which would induce oxidative stress to harm biomolecules of cells like proteins and DNA [36]. Thus, in order to further test the low toxicity of MoS_2_, PEGylated MoS_2_, and MoS_2_-PEG-PEI, intracellular ROS levels were assessed using a dihydroethidine (DHE) probe. In accord with the MTT result, no notable increase in the percentage of DHE-positive cells was observed for cells treated with MoS_2_, PEGylated MoS_2_, or MoS_2_-PEG-PEI for 24 h, suggesting minimal oxidative stress induced by those nanosheets (Figure 3d). Low toxicity of MoS_2_ nanosheets is ensured to further explore the nanomaterials as a drug carrier.

Polo-like kinase 1 (PLK1) was reported to be crucial in DNA replication [37,38]. And it was always overexpressed in many types of cancer cells [39]. The silencing of PLK1 would trigger cell apoptosis. Herein, we would like to use our MoS_2_-PEG-PEI as a nanovector for the delivery of PLK1 siRNA in order to test the transfection efficiency. To study the siRNA binding ability of our nanoparticles, we mixed MoS_2_-PEG-PEI with siRNA at different N/P ratios and carried out an agarose gel electrophoresis (AGE) assay (Additional file 2: Figure S2). Concluded from the AGE result, when MoS_2_-PEG-PEI was mixed with siRNA at N/P ratio above 5, significant retardation of siRNA movement in gel electrophoresis was observed. In contrast, bare siRNA could not be retarded.

**Figure 4:**
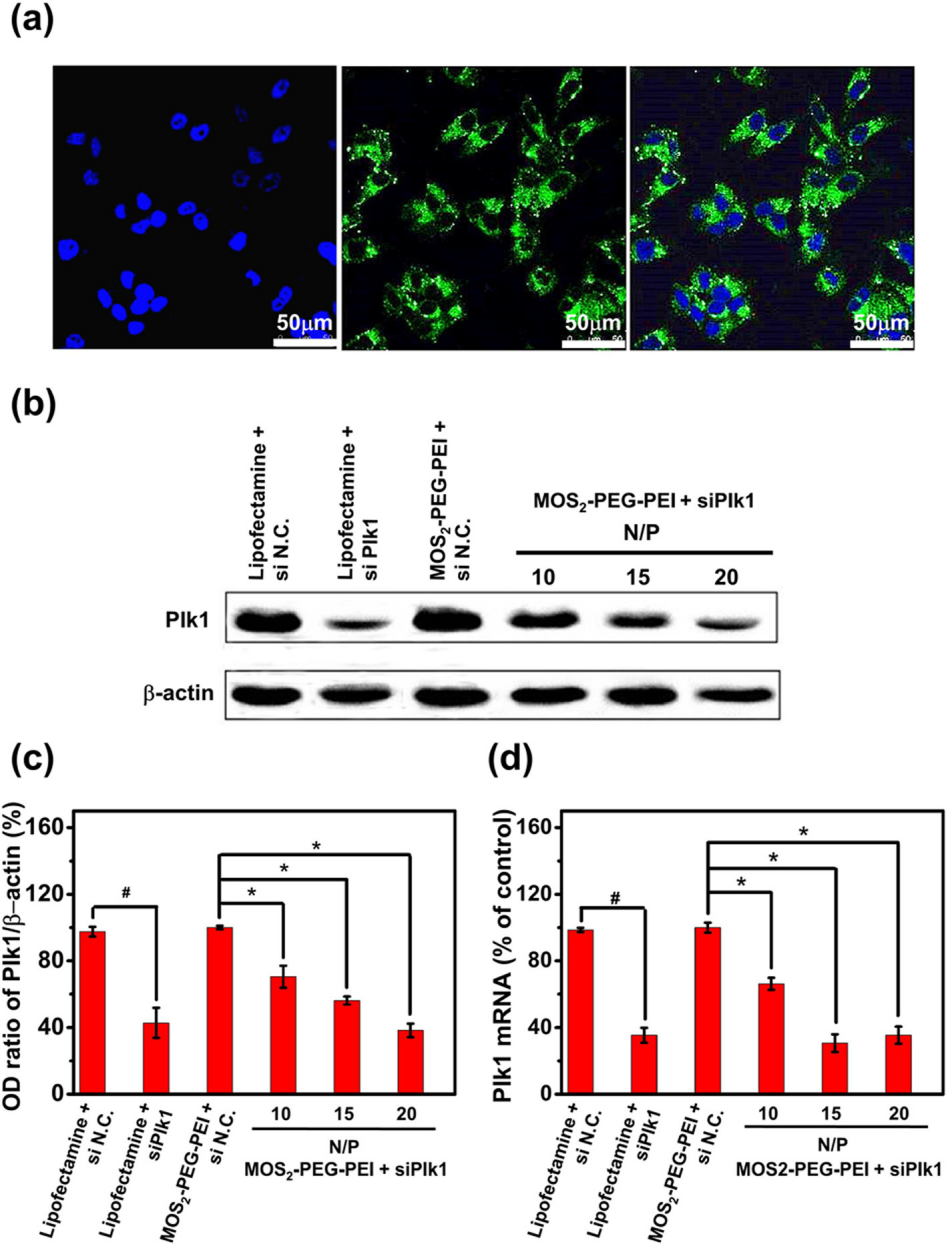
**Cell uptake and *in vitro *siRNA transfection. (a)** Confocal microscopy images of HepG2 cells after incubation with MoS_2_–PEG–PEI/FAM-siRNA for 4 h. The fluorescence from DAPI (blue colored) and FAM-siRNA fluorescence (green colored) showed well co-localization inside cells. **(b)** Western blotting results to determine PLK1 expression of HepG2 cells after various treatments indicated. β-Actin was also detected as the internal control. **(c)** Quantitative determination of PLK1 expression for different samples based on Western blotting data from (b). **(d)** The expression levels of PLK1 mRNA determined by qPCR. PLK1 mRNA levels were expressed as a relative index normalized against β-actin. Error bars were based on triplicated samples. *P* values were calculated by the Student’s *t*-test: ∗, #*p* <0.05 (*n* =3).

We next used confocal fluorescence microscope to study the cellular uptake of MoS_2_-PEG-PEI/siRNA complex (Figure 4a). The fluorescent signal from DAPI and FAM-labeled siRNA were simultaneously detected. Clear co-localization of signals from two different channels was detected, suggesting that siRNA was successfully shuttled into cells by MoS_2_ nanosheets.

To determine the expression of PLK1 after MoS_2_-induced siRNA transfection, qPCR and Western blotting was conducted (Figure 4b,c). The qPCR result showed that lipofectamine-mediated transfection of siPLK1 (Figure 4b,c) led to a notable decrease of PLK1 compared to the control group (siN.C.). For cells treated with MoS_2_-PEG-PEI/siPLK1, obviously decreased PLK1 expression was observed with the increase of N/P ratio. In consistence with the qPCR result, Semi-quantification data of Western blotting (Figure 4d) uncovered that the PLK1 silencing efficiency with MoS_2_-PEG-PEI/siPLK1 was achieved as well as that with Lipofectamine 2000 at N/P ratio of 20, which indicated MoS_2_ as an effective transfection carrier.

**Figure 5:**
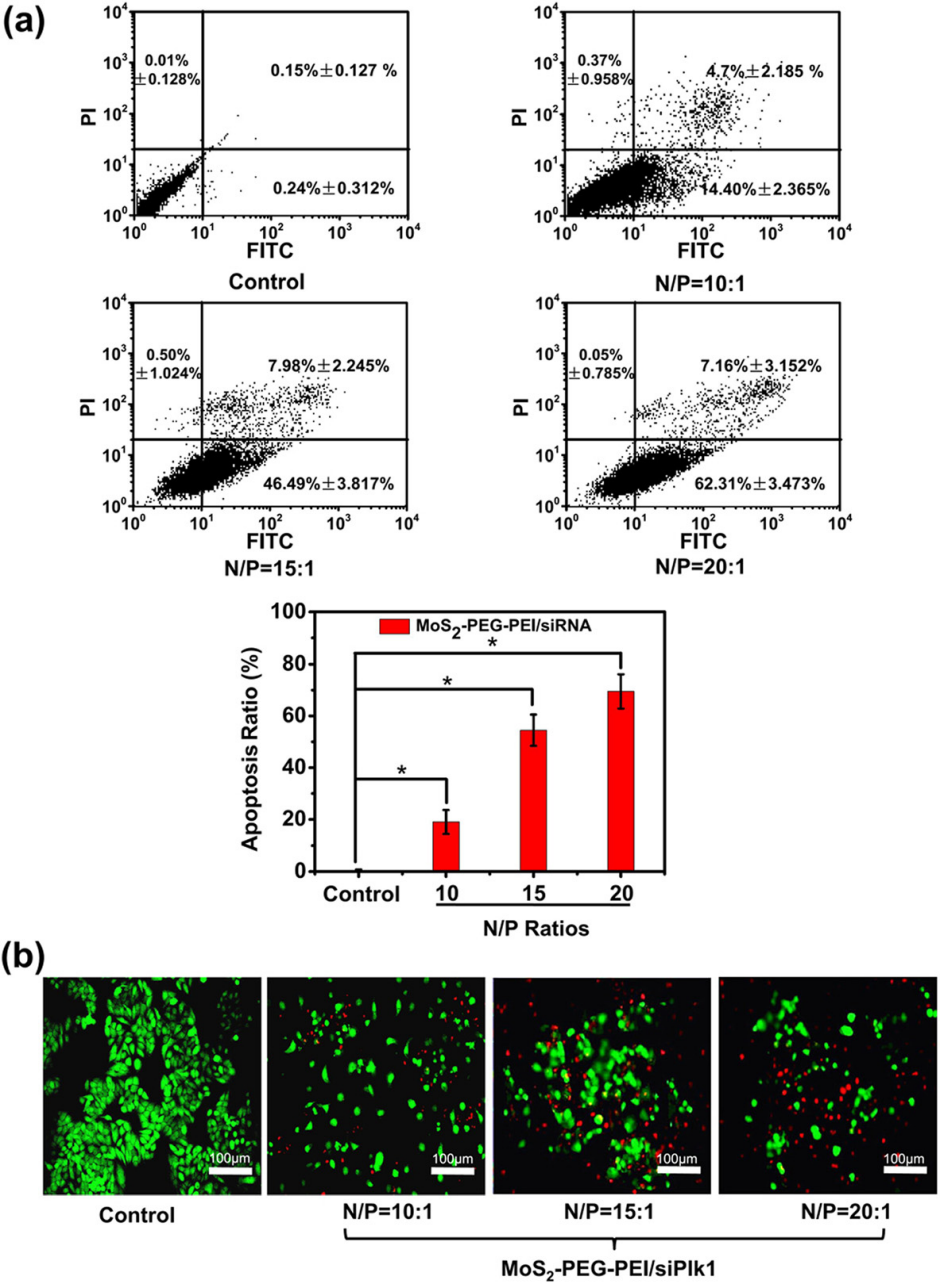
***In vitro *RNAi-induced cancer therapy. (a)** Flow cytometry analysis data of HepG2 cells after being treated. **(b)** Fluorescence micrographs showing the calcein-AM (green, for living cells) and PI (red, for dead cells) double-stained HepG2 cells. Scale bar: 100 μm. Error bars in (a) is based on triplicated samples. *P* values were calculated by the Student’s *t*-test: ∗p <0.05 (*n* =3).

It was documented that PLK1, as an oncogene, would trigger cell apoptosis when downregulated in cancer cells *in vitro*[40]. Thus, to further ensure the onset of the MoS_2_ and the role of siPLK1 transfection, flow cytometry analysis was employed to quantify the cell apoptosis post treatment (Figure 5a). The results showed that an increasing proportion of apoptotic cells treated with MoS_2_-PEG-PEI/siPLK1 were detected with the increase of N/P ratio. Microscopy images of calcein-AM and PI double-stained (living and dead cells) HepG2 cells also showed an increasing proportion of apoptotic cells treated with MoS_2_-PEG-PEI/siPLK1, which had a good accordance with the flow cytometry result (Figure 5b). These results further confirmed that MoS_2_-PEG-PEI acted as a kind of perfect nanocarrier for gene delivery.

The success of gene therapy mostly relies on the development of the gene delivery vector [41]. Currently, gene delivery systems are mainly categorized into viral and non-viral groups. In terms of the viral system, target genes can be packed into a virus like adenovirus, which has the capacity to inject its DNA into the host cells. However, the side effects of viral carriers, such as recombinant viral vectors reverting to their original wild type or the possibility of adverse immune responses to the host, limited their further application [42,43]. In contrast, non-viral vectors should circumvent some of the problems occurring with the viral vectors. Moreover, non-viral vectors have advantages in simplicity of use, ease of large-scale production [44].

In our present study, we utilized the MoS_2_-based material as gene carrier. Notably, the current results exhibited TMDCs as a novel type of 2D nanovector in gene delivery with low cytotoxicity and high transfection efficiency, promising for future applications in non-viral based gene therapy. Compared with many other widely explored agents, MoS_2_ was an essential trace element of life. Besides, our 2D MoS_2_-PEG-PEI nanosheets suggest no obvious cytotoxicity. However, although preliminary *in vitro* experiments suggest no obvious cytotoxicity of MoS_2_-PEG nanosheets, we should still keep in mind that the factors controlling the pharmacokinetics and biodistribution of non-viral vectors were complicated *in vivo*. More deep studies were required to understand the potential toxicity as well as possible metabolism of this type of TMDC material *in vivo*. Thus, more attention and effort should be taken to make the perfect gene carrier suitable for clinical use.

## Conclusions

Our work opened a novel and exciting avenue in gene delivery system. Combined with the advantages of high transfection efficiency of PEI, a new MoS_2_-based gene vector was successfully developed for gene therapy of cancer.

For the first time, MoS_2_ nanosheets with appropriate surface modification of PEG and PEI could be employed as a novel class of 2D nanocarriers for efficient siRNA delivery. And it was found that the MoS_2_-PEG-PEI nanosheets exhibited no observable toxicity to cells at the tested concentrations. Utilizing such a nanocarrier for PLK1 silencing, we achieved optimal gene knockdown and cancer cell apoptosis with N/P ratio of 20. Therefore, our study demonstrated that MoS_2_ with a well designed and engineered surface could serve as a nanocarrier that offered the novel opportunities in biomedical therapy [45].

## Competing interests

The authors declare that they have no competing interests.

## Authors’ contributions

ZK and XW generated the research idea, analyzed the data, and wrote the paper. RY and HC were involved in some of the sample preparation and material synthesis. QZ and LG performed the statistical analysis. BW and ZG provided the samples. XX, WC, and LG have given final approval of the version to be published. All authors read and approved the final manuscript.

## Supplementary Material

Additional file 1: Figure S1 MoS_2_-PEG-PEI stability. **(a)** MoS_2_-PEG-PEI stability in water, saline, and serum-containing cell medium at room temperature. **(b)** MoS_2_-PEG-PEI kept their consistent hydrodynamic sizes at about 50 nm in the serum-containing cell medium. Click here for file

Additional file 2: Figure S2 Gel retardation assay. Agarose gel electrophoresis of bare siRNA, MoS2 nanosheets, and mixtures of MoS2-PEG-PEI and siRNA at different N/P ratios. Each sample was incubated at room temperature for 20 min before electrophoresis. Click here for file
