# Supplementary figures and images for: A promising gene delivery system developed from PEGylated MoS2 nanosheets for gene therapy
Source: Nanoscale Res Lett. 2014 Oct 27;9(1):587. doi: 10.1186/1556-276X-9-587 (PMC4216190; doi:10.1186/1556-276X-9-587)

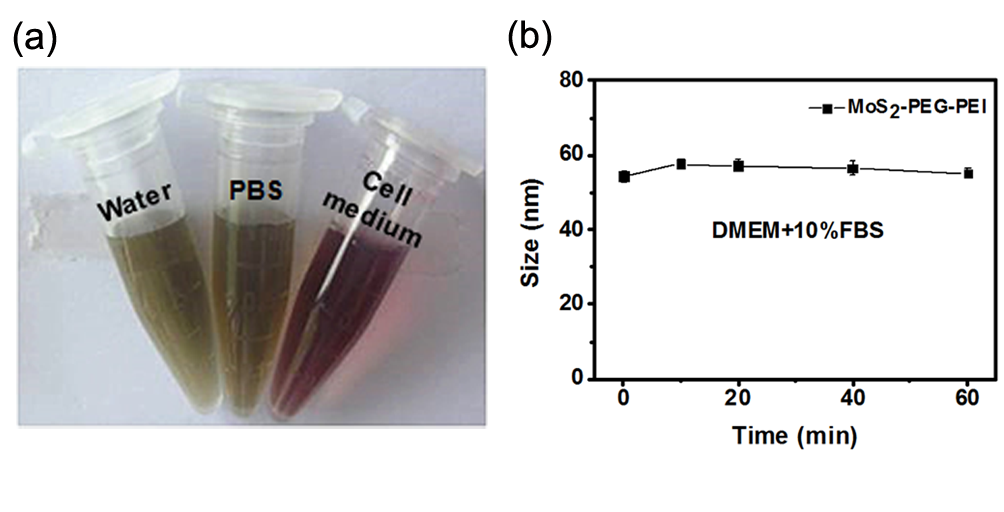

Supplement: Additional file 1: Figure S1 — MoS2-PEG-PEI stability. (a) MoS2-PEG-PEI stability in water, saline, and serum-containing cell medium at room temperature. (b) MoS2-PEG-PEI kept their consistent hydrodynamic sizes at about 50 nm in the serum-containing cell medium. [file 1556-276X-9-587-S1.tiff]

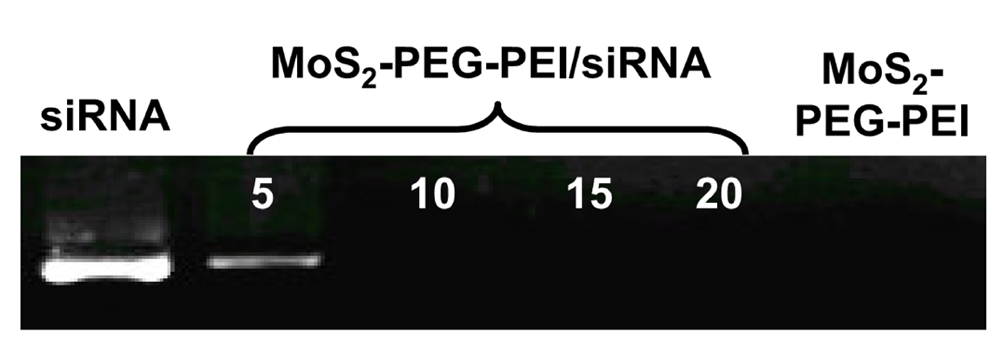

Supplement: Additional file 2: Figure S2 — Gel retardation assay. Agarose gel electrophoresis of bare siRNA, MoS2 nanosheets, and mixtures of MoS2-PEG-PEI and siRNA at different N/P ratios. Each sample was incubated at room temperature for 20 min before electrophoresis. [file 1556-276X-9-587-S2.tiff]
